# Human (in)security and psychological well‐being in Palestinian children living amidst military violence: A qualitative participatory research using interactive maps

**DOI:** 10.1111/cch.12917

**Published:** 2021-11-08

**Authors:** Guido Veronese, Federica Cavazzoni, Alec Fiorini, Hala Shoman, Cindy Sousa

**Affiliations:** ^1^ Department of Human Sciences “R. Massa” University of Milano‐Bicocca Milan Italy; ^2^ John Hopkins University Baltimore Maryland USA; ^3^ Newcastle University Newcastle upon Tyne UK; ^4^ Bryn Mawr College Bryn Mawr Pennsylvania USA

**Keywords:** human insecurity, Palestinian children, psychological well‐being, violence, war

## Abstract

**Background:**

Research has widely evidenced the effects of war and political violence on the functioning of children, with a great accord in diagnosing children's psychological burdens related to their exposure to violence. Yet, within this literature, the influence of the chronic sense of insecurity on their psychological functioning during and after hostilities remains unexplored.

**Methods:**

The present study aimed at exploring interrelated relationships between the perceived insecurity and the children's psychological well‐being and their adjustment to trauma. Based on drawings and walk‐along interviews with 75 Palestinian children, residents of both the West Bank and Gaza Strip, we offer an analysis of human security‐related risks and protective factors that contribute to either promoting or undermining the child's psychological functioning in a context characterized by chronic instability and political violence.

**Results:**

A complex network of sources of security and insecurity emerged from the narratives depicting an ecological portrait encompassing the determinants of children's mental health and psychological functioning. The TCA led to the identification of eight main themes: *school and associativism*; *social relations and house as a source of security/insecurity*; *military occupation as a source of insecurity*; *national and political identity as a source of safety*; *mosque and spirituality as a source of safety/unsafety*; *environment as a source of security/insecurity*; and *mental health*.

**Discussion:**

An approach encompassing human security as an explicative model can help in exhaustively portraying the complexity of the Palestinian children's suffering and their competence in adjusting to their traumatic reality. The study draws attention to social, political, environmental and economic determinants of children psychological well‐being.

**Key messages**

- Children in Palestine experience high degrees of human insecurity.
- Human insecurity affects children's mental health and psychological functioning.
- Individual and collective factors shape children's suffering in Palestine.
- Clinical work focused on adapting victims to abnormal living condition is ethically questionable and ineffective.
- Participative and community‐oriented programmes can generate healing forces within the Palestinian children and population.

## 1 INTRODUCTION

Studies have reported psychological burdens as a long‐term effect of children's exposure to armed conflicts and structural violence (Catani, 2018; Denov & Fennig, 2020). Most of the research associated the children's suffering during the war and their exposure to traumatic experiences with resultant trauma‐related pathologies (El‐Khodary et al., 2020; Karam et al., 2019), whereas social and political determinants of the war‐affected children's psychological breakdown have been much less considered and under‐analysed within the literature (Bloom, 2019; Dawes, 2020). In fact, the psychiatric discourse on war and violence is the dominant one in the mainstream clinical and health pathology (Summerfield, 1999, 2000).

A systematic review of 7920 children's mental conditions in the aftermath of war reported that post‐traumatic stress disorder (PTSD) was the primary syndrome, whereas in fewer studies, both elevated levels of depression and anxiety disorders were reported (Attanayake et al., 2009). In Gaza, 535 out of 549 children were found to suffer from some trauma‐related syndromes (El‐Khodary et al., 2020), whereas Syrian refugee children resettled in Jordan and Lebanon were diagnosed with PTSD associated with emotional dysregulation. Hence, a massive corpus of research has certified that PTSD syndromes are an epidemic among war and systematic violence‐affected children worldwide (Fazel & Stein, 2002; Khamis, 2012). Despite this, human rights‐related issues and their effect on children continue to be more volatile and controversial topics in the scientific arena (Denov & Fennig, 2020; Kienzler, 2019).

However, several sources of human insecurity have been identified as risk factors for children experiencing war and political violence. These factors comprise both person‐related and context‐related levels.

Because the United Nations Development Program (UNDP) (1994) set out an agenda on safety and development expanding the concept of human insecurity, scholars agreed that such a concept was strictly related to factors undermining the collective and personal well‐being of people affected by war, structural violence and chronic poverty (Ostergard & Griffin, 2019). McNeely et al. (2014) found a series of factors affecting the psychosocial functioning of victims of war in Palestine, which included political, economic and health‐related factors. These results confirmed Batniji and colleagues' analysis of the deteriorating living conditions in Palestine in the last 10 years because of a worsening and harsher military occupation that is restricting access to primary resources and limiting freedom of movement within, across and outside the occupied territories (Batniji et al., 2009).

Furthermore, Panter‐Brick and colleagues observed that human insecurity affected children's mental health in a refugee camp in the Middle East, hosting Syrian refugee children escaping from war and systematic violence (Panter‐Brick et al., 2018).

Thus, in order to address the gap in the literature in detecting the relationship between perceived insecurity and child psychological well‐being and adjustment to trauma, our paper aimed at qualitatively assessing human security‐related risk and protective factors that can contribute to promoting or undermining the child's psychological functioning in a context characterized by chronic instability, low‐intensity warfare and political violence.

## 2 METHODS

### 2.1 Participants

The participants were 75 children, all attending primary school. The children's ages ranged from 7 to 13 years old (M = 10.27; SD = 1.38), 51 females (68%) and 24 males (32%). Participants were chosen through convenience sampling, and data was collected between April to December 2018.

Children were recruited via well‐reputed local non‐governmental organizations working on children in cities, villages and refugee camps in Gaza and the West Bank of the occupied Palestinian territory (oPt). Inclusion criteria were living in the refugee camp, not being diagnosed with physical or psychological diseases and attending UN‐run school inside the camp. Inclusion criteria were related to both age and not being previously diagnosed with any physical or psychological diseases. Basic demographics were collected, including gender, age, site of residence and religious affiliation. Table 1 shows the distribution of the study sample, according to both site and gender.

**TABLE 1:** Distribution of the study sample

Area	Site	Setting type	Male	Female	Total
West Bank	Nablus	City	4 (5.3%)	11 (14.6%)	15 (20%)
	Dheisheh	Refugee camp	5 (6.6%)	24 (32%)	29 (38.6%)
	Fasayel village	Village	5 (6.6%)	9 (12%)	14 (18.6%)
Gaza Strip	Gaza City	City	3 (4%)	3 (4%)	6 (8%)
	Jabalia	Refugee camp	7 (9.3%)	4 (5.3%)	11 (14.6%)
			24 (32%)	51 (68%)	75 (100%)

### 2.2 Instruments and procedures

All the children were asked to draw a map on an A3 white paper representing all of the significant places in their neighbourhoods, whether they perceived them as safe or unsafe, and describe them. Participants were given three colours: green to represent safe places, black for neutral ones and red to represent unsafe places (Blaut et al., 2003). Upon completion of the drawing task (all children participated), 40% of them (seven from Nablus City, six from the village of Fasayel, 10 from Dheisheh refugee camp, three from Gaza City and four from Jabalia refugee camp) were invited to continue the interview during a walk‐around in their neighbourhood while showing the places they inhabit in their daily lives (Anderson, 2004; Carpiano, 2009). The remaining children (60%) did not participate due to previous commitments with their family, work or school commitments. The activity consisted of one child at a time leading the team on a tour of his/her neighbourhood, both among the places drawn and beyond them. This technique of ‘talking while walking’ (Anderson, 2004) has been described as a ‘walk‐around’ (Carpiano, 2009), and it has been specifically used to study the interactions and implications of place on people's general health and well‐being (Carpiano, 2009).

Participants were fully informed about the research aims, and all children and their families were asked to provide verbal informed consent. Children were free not to answer any of the researchers' questions or to withdraw themselves from the study at any moment. The research was conducted according to American Psychological Association (APA) ethical guidelines concerning child protection (APA, 2013) and approved by Milano‐Bicocca's Institutional Review Board (N.368).

All children's narratives were recorded, transcribed and translated into English by a local bilingual researcher and analysed by two independent researchers (Lambert, 2019). A deductive, top‐down content analysis approach was used to categorize data using NVivo12 Software. Inter‐rater agreement was satisfactory at 72%. The main themes were selected via a consensus by discussion procedure.

## 3 FINDINGS

The TCA led to the identification of eight main themes. Up to seven of the themes were declined regarding self‐perceived security and insecurity among the interviewed children. The remaining theme—*mental health*—was made of two subcomponents (*negative emotions and perceived insecurity tied to ongoing hardships* and *fear and traumatic memories*), explaining children's mental distress exposed to systematic violence and political oppression.

### 3.1 School and associativism as a source of security/insecurity

Education is an asset for people living under oppression, and school is also a privileged arena for establishing social connections and struggling for collective liberation (Reynolds, 2007; Todd, 2011; Vollhardt et al., 2020). As a consequence of the ongoing colonial and military violence, the school can be either protective or unsafe when disrupted by highly traumatic events (Marie et al., 2018).

School can be perceived as one of the few safe places to play and spend time socializing and learning in an occupied landscape. It is a shelter for an activity that can restore a sense of efficacy and endurance despite the surrounding uncertainty and instability (Veronese et al., 2015).

If there is bombing while we are in school, we do not get afraid. I feel safe at school. We are inside, and I am not afraid since I am with my friends. (Gaza, Y, 11 years old, male)

Paulo Freire explains in *Pedagogy of the Oppressed* (Giroux, 2010) that school is the privileged place for liberatory education. Accordingly, education is a practice of freedom and resistance in the face of despair and hopelessness that can allow children to perceive a sense of competence and endurance in a dangerous and disruptive environment. Education is a means to understand and resist oppressive and to colonize structures of power that dehumanize the Palestinian community and subjugate any attempt of a critical and anti‐colonial interpretation of historical facts in the region, ultimately creating a system of repression and surveillance that undermines people's well‐being and functioning (Veronese, Sousa, et al., 2020).

I want to study, be educated to become a cop. A cop because I want to save my country, to make it safer. I want to expel the occupation and be free and in peace. (H, male, 12 years old, Dheisheh camp)

Likewise, girls think of their education as an opportunity for gender‐informed practices that can challenge masculine norms and provide women with opportunities to contribute to the political liberation and social transformation of their country while also improving their mental well‐being and challenging the patriarchal discourse of power that pervades the Palestinian society (Shalhoub‐Kevorkian, 2008).

Education is crucial for me […]. It is important for women; they can get a chance. In this community, man is everything […]. Women should take some of the power that men have. Women are not safe because they do not have rights. With education, they can improve their right. Education is important because women can have the chance to change their conditions. (M, female, 13 years old, Dheisheh camp)

On the contrary, school and associative places can be perceived as unsafe or dangerous spaces exposed to military incursions, intra‐community violence and aggression. Furthermore, there is a risk of school being negatively perceived as an oppressive institution where pupils feel overwhelmed by their duties (e.g. school assignments and institutional pressures) and frightened by unhealthy relationships with teachers and adults (Elbedour et al., 1997; Kortam, 2018).

Structural violence contributes to spreading a diffuse sense of unpredictability that children can perceive in their ordinary school life, thereby transforming positively connotated places that are universally perceived as protective into uncertain places that are out of control, permeable to violence and repression. In so doing, actions aimed at terrifying and annihilating potential threats to the colonial status quo are more than occasionally perpetrated under the justification of preserving Israel's security (Pherali & Turner, 2018).

I usually feel safe in the school, but not always. Do you know why? The Israeli army once invaded our school. They stopped students on that day. (H, male, 10 years old, Dheisheh camp)

Structural and systematic violence can undermine a crucial protective factor for Palestinian children, such as school and education, transforming it into a site of insecurity that fosters negative emotions and psychological suffering (Veronese et al., 2017).

Children reported peer violence as a source of insecurity and school as a place where children can be at risk of aggression by their schoolmates, including older boys. In an environment characterized by oppression and lack of freedom, these aggressive behaviours are not usually perceived as exceptional by the children but rather as everyday practices within a social environment diffused by dispossession and trauma (Akesson, 2015).

I feel unsafe in my school because [older] boys hit me. (Q, Nablus, 10 years old, male)

I do not like because they take my ball. Boys are not nice in my school and violent. (H, 10 years old, male, Gaza)

Consequently, adults themselves can make school a source of insecurity that can negatively impact the children's development and functioning.

Teachers punish and hit. Sometimes they dismiss students who do not wear the uniform. I do not like this; they are bad in this. I feel afraid when I see this. (E., female, 9 years old, Dheisheh camp)

Punishment and suppression of freedoms can be considered the mark of a fragmented society where social suffering shapes individual and collective experiences.

I do not like when the headmistress gets angry and shouts at us. Also, I would not say I like school when fights happen because fights cause chaos. I am not too fond of it; chaos makes me scared and confused. I do not like these problems with the fight. When they make troubles, and the social specialist cannot solve them. (Yasmin, female, 11 years old, Fasayel)

### 3.2 Social relations as a source of security/insecurity

Sociality in a collectivistic society can be counted as a protective factor from hardships and violence (Afana et al., 2018). Sharing experiences and emotions within the family circle, both nuclear and extended, can provide children with adjustment and coping strategies and reduce their sense of isolation and despair due to the unstable and disrupted living environment (Srour & Srour, 2006).

I go to school with my brother, he can help so that no one can hit me. When we are two, together, I feel that we are stronger than others are. I feel strong and happy. (Q., 10 years old, male, Nablus)

Within the family, children assume various developmental tasks that maintain their connection to the family unit and guarantee emotional and cognitive containment amidst hard times in a traumatic reality (Kankaanpää et al., 2020).

I feel safe when my family is at home, when they are not in the house I do not feel safe. My family makes the house safe, not really the house itself. (A, male, 11 years old, Dheisheh camp)

On the other hand, strict social control within the family creates a sense of social suffocation that prevents individuation, especially among the young generation, including children (Akesson & Grinberg, 2020). A lack of individual freedom is a consequence of being controlled and judged by family members, both at a nuclear and extended level (Kortam, 2018).

In those houses, there is a relative who fought with my mother, and her sister does not want to talk with my mother. A third one kept coming to our house, but my mother does not talk with her anymore. I do not feel good here with them. (N, 10 years old, male, Nablus)

However, community members are perceived as a source of security when neighbours contribute to making a living environment that can be better understood and negotiated by children, facilitating warm relationships and accessibility to protective places (Rabaia et al., 2018).

People are nice there, and they help me. I feel good. If I lose myself, I have known that going there I will find people to help me. (T, female, 11 years old, Nablus)

However, collective trauma and societal fragmentation can lead to internal fights that increase distrust, social concern and a sense of precarity among children.

I could be kidnapped in this place. I feel it is not safe because I do not know people here, and there are many strangers. They might be kidnappers. Also, owner of shops may harm me. (R, female, 10 years old, Dheisheh camp)

A loss of trust and confidence in people risks turning the protective power of social behaviour into a source of insecurity that augments children's feeling of mental distress (Thoresen et al., 2018).

When I was little, boys were beating me and mocking me here in the neighbourhood. I do not know why. I was so afraid. I am afraid until now. I do not come here alone, or I do, but I try to make it very brief. (K, male, 10 years old, Nablus)

### 3.3 House as a source of security/insecurity

A house can be considered the most quintessential site of protection and safety for children, especially when we analyse a society organized and shaped by familial norms (Kulwicki, 2020). Children describe their homes as a place of emotional nurturing and growth, as well as an emotional safe space protecting children from both fear and a sense of danger in the wake of military incursions and attacks.

It is very safe my house. They [the Israeli Army] never entered in my house. I can sleep because it is safe. (H, 10 years old, male, Dheisheh camp)

Home is a medium for promoting positive affects where children locate the foundation for healthy social development and positive psychological functioning (Massad et al., 2018). Despite the home being one of the most critical and salutogenic places in Palestine, it can also generate many conflicting meanings. For example, children can also experience the home as a source of uncertainty and anxiety (Akesson, 2014). Palestine has been described as a country characterized by ‘domicide,’ which is a consequence of the settler colonialist violence of the neighbouring and occupying Israeli state (Akesson et al., 2016). Most families experienced direct or indirect violations, including damage or destruction to their houses that render a safe space into a site of danger and uncertainty. In this place, children cannot be adequately protected from pervasive and violent external forces.

In fact, the Israeli occupation and ongoing military aggression is making the home's boundaries more permeable and out of the dwellers' control. The extension of instability and danger into the family home increases fear, anxiety and a sense of uncertainty among Palestinian children.

They [soldiers] are terrifying and dangerous. They came during the night, and they arrested my brother. I was terrified, and I went to stay with my mother. Since then, I am always very afraid when they come, and I go to my mother's room. (A, female, 7 years old, Dheisheh camp)

As previously described, military violence systematically targets the symbolic places of security, thereby spreading terror and panic within the civilian population to annihilate any attempt of psychologically or physically resisting the colonial domination and administration of their native lands (Joronen & Griffiths, 2019).

### 3.4 Military occupation as a source of insecurity

The Palestinian landscape is marked by signs of structural and organized violence (Weitzman, 2007). Living within this traumatic reality endangers childhood growth and development as they must constantly adjust to abnormal living conditions, comprising soldiers' harassment, settler violence, humiliating checkpoints, a separation wall and fences and barriers that jeopardize the Palestinian territory. This sense of social suffocation and hopelessness among Palestinian children is the direct psychological consequence of Israeli military surveillance (Khamis, 2020).

This is the fence; there are Israeli. It is dangerous because Israelis are here, plant landmines, fire people, and shells. […] I am afraid of this place because there are Israeli and fires. I am afraid of landmines since they can explode around us, and I am afraid of fires, tanks shell, and warplanes because they launch rockets. (Y, 11 years old, male, Gaza)

In such a way, children perceive their social existence as under constant risk of material dispossession, where spaces are reshaped by the presence of a military power that prevents freedom of movement and circulation and prohibits self‐determination under the threat of military incursions.

Sometimes we get on a ship, but we do not sail far away because it is dangerous for the sea and the Israeli. I want to sail far away, but we will finish the space the occupation gave us if we do this. They give us a space; we cannot go more than that, or it is dangerous. (Y, 11 years old, male, Gaza)

### 3.5 National and political identity as a source of safety

Security can be perceived at a macro level through the national sense of belonging, fostering feelings of protection and self‐esteem among children. National identity can be considered a source of security and political well‐being (Veronese, Pepe, et al., 2020). In fact, active participation in the struggle to end the occupation provides children with a sense of social competence and agency within an otherwise hostile and constraining environment.

Here I put a Palestinian Flag. I feel safer when I see it. Because our houses are in Palestine. And seeing the flag, we all know that this is Palestine. (H, female, 11 years old, Fasayel)

Symbols such as the national flag or traditional dress serve as protective factors and sources for promoting self‐esteem.

It is a symbol of Palestine [the Palestinian dress]. I like all people here in Palestine, and I feel safe here. We should always remember our country. I like to wear this, to feel Palestinian, and this makes me feel so good. (H, female, 11 years old, Fasayel)

Traditional habits and collective ceremonies are sources of security and a platform for mental well‐being among children searching for a national identity, disrupted by a multigenerational diaspora that has dispersed the Palestinian people all over the region and world.

Dabka forms my Palestinian identity. It is resistance for me. Again resistance is not only by stones and weapons but also by dancing dabka. I feel safe and strong when I am here dancing. I feel proud of my identity. (M, female, 13 years old, Dheisheh camp)

### 3.6 Mosque and spirituality as a source of safety/danger

Mosques and other religious places are perceived as safe spaces where children can cultivate spiritual well‐being and happiness. Religious events are equated with liberated spaces where children can cultivate a Muslim identity that extends beyond national borders and regain a sense of control over their lives and hope and confidence for a safer future.

This place is essential for me. I love the mosque because here I can feel so comfortable. After all, this is the house of God. I feel tranquil and quiet when I enter here because this is the house of God, and there are Shaikhs here. (S, 10 years old, male, Gaza)

However, religious events can also be associated with traumatic experiences, such as violent demonstrations (i.e. the great march of return in Gaza) where people are at risk of being shot dead by Israeli snipers (Mills et al., 2019).

I hear the mosques when they call people to go to the Great Return March. Everybody can hear it. I am afraid of it, and I do not like it. I wish they stop to call. (Y, male, 11 years old, Gaza)

### 3.7 Environment as a source of security/insecurity

Children tend to perceive outdoor places and the surrounding environment as a source of insecurity, particularly when these spaces are associated with remote, abandoned, unclean areas or crowded and polluted streets. Such a sense of insecurity reveals an environmental crisis in Palestine that puts children's health and mental well‐being at risk and reduces opportunities for activities and free movement within the occupied territories (Baraquoni et al., 2020)

I feel very scared here because it is full of rats. Q, male, 10 years old, Nablus

We do not play outside. It is dangerous because when we play outside, cars, jeeps go back and forth. I am not afraid of cars, but here it is dangerous, no space for people, accident with cars have happened. (H, female, 11 years old, Fasayel)

Living in an unsafe and disrupted environment impacts children's life satisfaction, especially because it prevents them from actively transforming and ameliorating their surroundings (Veronese, Dhaouadi, et al., 2020).

I do not feel safe there; it is full of rubbish. There is no dustman or container to keep the environment clean. When I see garbage, I feel depressed. […] This is the most thing I hate, the rubbish and I am afraid of it because weasels and dog gather around it. (HJ, 12 years old, male, Dheisheh camp)

Accordingly, unknown and remote places are perceived as dangerous and unsafe.

I do not feel safe in the place that is far. I do not know people there, and they do not know me. (J., 10, female, Nablus)

On the other hand, well‐known places contribute to fostering children's self‐confidence and sense of security.

I feel safe here because I was born here, I know this place. It is nice to explore places outside, but the best place is the place where you were born. Here I feel safe. (A, 13 years old, female, Nablus)

Parks and the few other available green areas also improve the children's perception of well‐being and satisfaction with their own lives as it helps them overcome the sense as mentioned above of social suffocation.

I feel thrilled watching natural things. Sometimes I shower under it. I drew trees, too. I feel my mind is relaxed when I see trees, and I feel peace. Nothing worries me. Also, I like the sun. It is a natural thing. (Y, male, 11 years old, Jabalya)

### 3.8 Mental health

The sources of insecurity mentioned earlier significantly impact children's psychological functioning and mental health (El‐Khodary & Samara, 2020). Negative affects are associated with traumatic experiences that increase children's mental distress and individual suffering in conditions characterized by ongoing social trauma.

I saw things like this on TV [Great March of Return clashes]. I feel sad, and I wish to kill other people instead of the occupation, like Arabs because they know what is happening, they watch, but they are not helping us. (Y, male, 11 years old, Jabalya camp)

Powerlessness and a pervasive sense of being unable to control surrounding spaces can trigger depressive thoughts and anxiety among children, as well as an enduring feeling of being under constant threat.

I really feel bad and depressed about the settlement [Jericho's settlement]. I do not like them. They arrested many people. They arrested my brother, and I am so sad about it. I am afraid every day that they will come back. If they bother me, I will not do anything, or they might react in a bad way. They have guns. (N, male, 10 years old, Dheisheh camp)

Sadness and fear undermine the psychological development of children exposed to multiple traumatic experiences, and it generates traumatic memories and acute stress (Peltonen et al., 2017).

Gaza is unsafe because suddenly you can be bombed by a rocket, so I do not like it. Sometimes I think about the bombing, but not always and I try to forget it. Sometimes it is difficult to forget it. (R, female, Gaza city, 11 years old)

War and structural violence also contribute to the overall traumatic reality in which Palestinian children try to adapt to the threatening social environment and overcome the psychological burdens placed upon them by the Israeli occupation (Giacaman, 2020).

Something happened here to me. That is why I do not like it. Our old house was here, and when wars broke out, something happened here. I was a little kid in the 2008 war; the Israelis stayed in our home. I was tiny, but I remember the fear. Since then, I do not want to stay in this area. (R, female, Gaza city, 11 years old)

Trauma is widespread among children in Palestine (Espié et al., 2009). The peculiarity of the complex trauma affecting the Palestinian population, including children, is marked by the absence of a ‘post’ condition. In fact, children are enmeshed in a distressing reality where the trauma is ongoing, multiple, multigenerational and historical (Mahamid, 2020; Mahamid & Veronese, 2020).

I hate the Israeli because they are bad, and they intimidate me. They came during the night, and they arrested my brother. I was terrified. Since then, I cannot stop being terrified when they come, I cannot sleep, and I go to my mother's room. (A, female, 7 years old, Dheisheh camp)

Chronic violence and direct exposure to the brutality of the Israeli army characterizes the children's lives, which creates a pervasive feeling of existential insecurity.

I am terrified from them. […] I feel afraid, when I remember the man Israeli army killed I cry, I cannot bear it. I imagine the blood of M. on the ground and when the Israeli army walked on the blood. (A, female, 8 years old, Dheisheh camp)

## 4 DISCUSSION AND CONCLUSION

**FIGURE 1:**
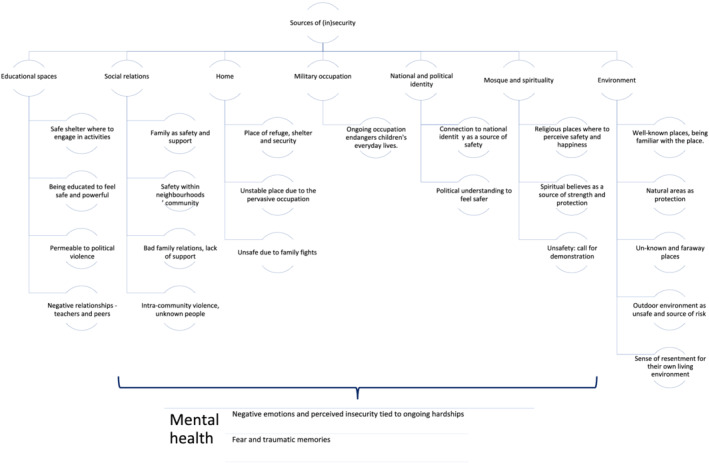
Sources of human (in)security emerging from children narratives

A complex network of social insecurity emerged from our participants' narratives, which depicted a social environment that determines children's mental health and psychological functioning. Within the Palestinian community, individual and collective factors shape children's suffering (Meari, 2015). Children perceive themselves as part of a community struggling for recognition and survival, overwhelmed by decades of oppressive colonial domination, dispossession and human insecurity (Figure 1).

Regarding the demographic variables, some gaps between living contexts and gender differences emerged from the analysis. Children living in refugee camps and villages were more exposed to the burdens of the Israeli military occupation than children living in the cities. Environmental barriers, greater exposure to military and settler violence bared those children as living a greater sense of insecurity than those living in urban areas (Cavazzoni et al., 2021; Mahamid & Veronese, 2020). Moreover, boys were more at risk of traumatic experiences than girls, most often more protected in the domestic sphere. However, girls seemed to perceive other forms of insecurity, more related to a form of patriarchal marginalization and relegation to the margin of the Palestinian community (Mahamid, 2020). We acknowledge that engendered forms of insecurity must be further studied and deeper discussed in future research.

A holistic approach to analysing Palestinian children's social suffering and their ability to adjust to their traumatic reality must encompass human security as an explicative model (Cavazzoni et al., 2020, 2021; Veronese & Cavazzoni, 2020). The social, political, environmental and economic determinants of children's psychological well‐being must be taken into consideration in approaching the assessment, diagnosis and therapeutic intervention, especially when we are confronted with environments characterized by structural violence and severe violations of human rights (Batniji et al., 2009; Ziadni et al., 2011). In Palestine, approaches exclusively oriented at alleviating the psychological burdens of the victims of systematic violence, as well as fostering resilience within such battered communities, must simultaneously be oriented at subverting and changing the underlying conditions that contribute to the maintenance of systems of oppression and violence (McNeely et al., 2014; Veronese & Castiglioni, 2015). On the other hand, clinical work‐oriented solely at reducing symptoms or adapting victims to abnormal living conditions, although ethically questionable, could reveal its inefficacy, if not its iatrogenic effects (Giacaman, 2014; Kienzler, 2008).

In contrast, participatory programmes can help Palestinian children challenge and subvert dominant power structures that oppress them. These programmes enable them to transform their surrounding environments and increase their resistance and endurance to traumatic circumstances, as well as restore a sense of security that has been undermined by decades of oppression and structural violence (Rabaia et al., 2014). Community‐oriented programs can generate healing forces within the Palestinian population that utilize political well‐being, a sense of collective belonging, and participation as protective dimensions to control human insecurity (Hammoudeh et al., 2013) and foster agentic behaviours in children.

A sensitive approach to human security frameworks must also consider children's mental health as a holistic and multifaceted dynamic process. Thus, psychological well‐being is made of interlocked domains and dimensions involving micro (individual's reactions to adverse conditions), miso (economic, political and environmental conditions affecting systems and acting within and between communities) and macro levels (spirituality, national identity and sense of belonging) (Gostin, 2001) that are promoting or impeding the sense of personal security of children affected by systematic violence and political oppression (Batniji et al., 2009).

Finally, some limitations to this study must be acknowledged and discussed. Firstly, the researchers engaged in this exploratory research were trained in a Western context and Westerners. This could have affected both the interaction and dialogue with indigenous children and the interpretation of the results. However, the diversity of the research team could have controlled such kinds of biases. Besides, and this could be considered a second limitation, short phase data collection could reduce the richness of the information and reflective elaboration of the participants' perspectives. For future research, ethnographic approaches and multicultural research teams are strongly recommended.

## Data Availability

Due to the sensitivity of the topics and potential risks for children, data are available only on request to authors and after careful consideration of the research team.
